# The effect of selective decontamination on the intestinal microbiota as measured with IS-pro: a taxonomic classification tool applicable for direct evaluation of intestinal microbiota in clinical routine

**DOI:** 10.1007/s10096-022-04483-8

**Published:** 2022-10-01

**Authors:** M. L. M. van Doorn-Schepens, G. S. A. Abis, S. J. Oosterling, M. van Egmond, L. Poort, H. B. A. C. Stockmann, H. J. Bonjer, P. H. M. Savelkoul, A. E. Budding

**Affiliations:** 1 Department of Medical Microbiology and Infection Control, Amsterdam UMC, Vrije Universiteit Amsterdam, De Boelelaan 1118, PK1X124, Amsterdam, 1081 HZ the Netherlands; 2 Laboratory of Medical Microbiology, Onze Lieve Vrouwe Gasthuis, Amsterdam, the Netherlands; 3 Department of Surgery, Amsterdam UMC, Vrije Universiteit Amsterdam, Amsterdam, the Netherlands; 4 Department of Surgery, Meander Medical Center, Amersfoort, the Netherlands; 5 Department of Surgery, Spaarne Gasthuis Hospital, Haarlem/Hoofddorp, The Netherlands; 6 Department of Molecular Cell Biology and Immunology, Amsterdam UMC, Vrije Universiteit Amsterdam, Amsterdam, the Netherlands; 7 Amsterdam Infection & Immunity Institute, Amsterdam, the Netherlands; 8 In Biome, Science Park, 116, Amsterdam, 1081 XG The Netherlands; 9 Department of Medical Microbiology, NUTRIM School of Nutrition and Translational Research in Metabolism, Maastricht University Medical Center, Maastricht, The Netherlands

**Keywords:** SDD, Microbiota, Colorectal cancer surgery, IS-pro

## Abstract

Selective decontamination of the digestive tract (SDD) is aimed at elimination of potential pathogenic microorganisms. In this study, the effect of SDD on gut microbiota was evaluated in a large homogenous group of elective colorectal cancer surgery patients. Rectal swabs were taken from 118 patients undergoing colorectal surgery. These patients were randomly assigned to receive perioperative SDD or to the control group (no SDD). Rectal swabs were taken prior to surgery, 3 days after commencing administration of SDD. Gut microbial profiles were obtained with the IS-pro technique, a standardized microbiota profiling assay applicable in clinical routine. Differences in abundance for different taxonomical groups and diversity between the groups were assessed. Unsupervised and supervised classification techniques were used to assess microbial signatures, differentiating between the SDD group and the control group. Patients in the SDD group had different gut microbial signatures than in the control group, also in phyla that are not a target for SDD. *Escherichia coli*, *Sutterella* spp., *Faecalibacterium prausnitzii*, and *Streptococcus* spp. were the species that differed the most between the two groups. The SDD group showed clustering into two subgroups. In one subgroup, a decrease in Proteobacteria was observed, whereas the other subgroup showed a shift in Proteobacteria species. This study shows that SDD not only decreases colonization of the gastrointestinal tract with potential pathogenic Gram-negative microorganisms, but also reduces the abundance of normal colonizers of our gastrointestinal system and leads to a shift in total microbiota composition.

## Introduction

On many intensive care units (ICUs) in the Netherlands, selective decontamination of the digestive tract (SDD) has been administered to patients for several decades. SDD decreases the rate of ventilator associated pneumonia [1] and ICU related mortality [2]. ICU-related infections are mainly caused by aerobic Gram-negative bacteria (e.g., *Escherichia coli*, *Klebsiella* spp*.*, *Proteus* spp*.*, *Pseudomonas aeruginosa*, *Enterobacter* spp.), *Staphylococcus aureus*, and yeasts [3]. These potentially pathogenic microorganisms (PPMs) commonly colonize the oropharynx and gastrointestinal tract of hospitalized patients. To prevent or eliminate colonization and subsequent infection with PPMs, SDD is administered in the oropharynx and gastrointestinal (GI) tract. SDD consists of a mixture of nonabsorbable tobramycin, colistin, and amphotericin B. This mixture is supposed to selectively eliminate PPMs while leaving the anaerobic intestinal microbiota intact [4–6].

There have been few studies that addressed the effect of SDD on gut microbiota. Studies based on microbial culture show that administering SDD leads to a decrease in the number of patients that are colonized by Gram-negative bacteria in the gastrointestinal tract [1]. However, the vast majority of our gut microbiota is not readily cultivable, which is why culture based studies yield biased results on the effects of SDD on gut microbiota. A limited number of studies based on molecular techniques has been performed to assess the effect of SDD on gut microbiota [7, 8]. Also, this limited number of molecular studies are based on small study groups, and have mainly evaluated gut microbiota composition with techniques such as quantitative polymerase chain reaction (QPCR) or fluorescent in situ hybridization (FISH) that target specific species.

Moreover, in both the culture- and molecular-based studies, patient groups were small and heterogeneous [7, 8]. ICU patients have different comorbidities and confounding factors that may influence gut microbiota composition, such as feeding through a nasogastric tube [9], sepsis [10], a range of medication which can affect gut microbiota [11], and a variety of antibiotics besides SDD which are commonly administered to these patients. Together, these factors can severely confound results when studying the effect of SDD on the composition of gut microbiota.

Here, we evaluated the effect of SDD on total gut microbiota, in a large homogenous group of elective colorectal cancer surgery patients, who were randomized to receive perioperative SDD or no SDD. We used the standardized IS-pro technique, which is a eubacterial molecular profiling assay based on ribosomal DNA signatures. The IS-pro technique is amenable to implementation in clinical routine, which makes our results directly translatable to diagnostic implementations.

## Methods

### Design

This study was accessory to the SELECT trial: Perioperative Selective Decontamination of the Digestive Tract (SDD) in Elective Colorectal Cancer Patients: a Multicenter Randomized Clinical Trial [12]. The SELECT trial is registered at ClinicalTrials.gov, identifier: NCT01740947. This descriptive, prospective accessory study was initiated by the Vrije Universiteit Medical Center (VUMC). Study subjects were randomly assigned to the intervention or control group. The intervention group (hereafter referred to as SDD group) received SDD four times daily, starting 3 days before surgery. SDD was administered orally and consisted of a 10-ml suspension containing 5-ml amphotericin B (500 mg) and 5 ml of a mixture of colistin (1000 mg) and tobramycin (80 mg). The control group did not receive SDD. Both groups routinely received a single preoperative parenteral dose of 1000 mg cefazolin and 500 mg metronidazole. Oral mechanical bowel preparation was given for left-sided colonic, sigmoid, and low anterior resections. A preoperative rectal swab was taken in the operating theater from all subjects.

### Subjects and clinical data collection

Colorectal carcinoma patients undergoing elective colorectal surgery (including construction of an anastomosis) with curative intent were recruited by the participating hospitals of the SELECT trial group. Inclusion and exclusion criteria and randomization have been extensively described in the study protocol [12]. Written informed consent was obtained from al study subjects. Furthermore, the study was approved by the Medical Ethical Review Committee of the VUMC and the Central Committee on Research Involving Human Subjects. The ethics approval was also stated in the SELECT trial, registered at ClinicalTrials.gov, identifier: NCT01740947.

Baseline characteristics including age, gender, smoking habits, body mass index (BMI), surgical history, tumor type, and preoperative radio (chemo) therapy were obtained.

### Rectal swabs

Rectal swabs provide a good method to produce highly reproducible microbiota profiles [13]. The rectal swabs (FLOQSwabs 552C, Copan, CA, USA) were taken preoperatively. Swab tips were transported in a sterile container which contained 500 µl reduced transport fluid (RTF) buffer. Within half an hour of transportation, they were stored at a temperature of −20 ºC, prior to sample handling.

### IS-profiling of the intestinal microbiota

Analysis on the intestinal microbiota was performed with the IS-profiling (IS-pro) technique, as described previously [14]. With IS-pro, bacterial species are discriminated based on the length of the 16S-23S rDNA interspace region in combination with phylum specific sequence variations of the 16S rDNA. The IS-pro technique consists of 3 separate steps, described more in detail below. To control for background noise, a negative control was taken along throughout the entire proces up to IS-pro analysis for every DNA extraction batch.

#### DNA isolation

DNA from the samples was isolated with the IVD-labeled, automated NucliSENS®easyMag® extraction system (easyMag, Biomerieux). One milliliter of lysis buffer including guanidine thiocyanate was added to each Eppendorf tube containing a swab. Tubes were vortexed for 5 min at 1400 rpm (Thermomixer comfort, Eppendorf, Hamburg, Germany). Hereafter, tubes were centrifuged for 2 min at 14000 rpm. Of the supernatant, 200 µl was added to the easyMag container, together with 2 ml of lysis buffer. After the mixture was incubated for at least 10 min at room temperature, DNA extraction was performed on the easyMag machine with the specific A protocol, as described by the manufacturer. The DNA was eluted in 110 µl buffer and stored at 4 °C prior to polymerase chain reaction (PCR) amplification.

#### IS-fragment amplification

IS-fragments were amplified in 2 separate PCR reactions, with phylum specific, fluorescent primers. In the first PCR reaction, IS-fragments of bacteria belonging to the phyla Firmicutes, Bacteroidetes, Actinobacteria, Fusobacteria, and Verrucomicrobia were amplified. In the second PCR reaction, IS-fragments of bacteria belonging to the phylum Proteobacteria were amplified. Amplifications were performed on a GeneAmp PCR system 9700 (Applied Biosystems, Foster City, CA). Cycling conditions for PCR were 35 cycles of 94 °C for 30 s, 56 ºC for 45 s and 72 ºC for 1 min; and a final extension at 72 ºC for 11 min.

#### IS-fragment analysis

After the PCR reactions were completed, 5 µl of PCR product was mixed with 19.5 µl formamide and 0.5 µl custom ROXlabeled size marker (BioVentures, Murfreesboro, TN, USA). Subsequently, DNA fragment analysis was performed on an ABI Prism 3500 Genetic Analyzer (Applied Biosystems). All data were preprocessed with the proprietary software suite (IS-Diagnostics, Amsterdam, the Netherlands) and further analyzed with the Spotfire software package (TIBCO, Palo Alto, CA, USA).

### Statistical analysis

After processing the data, each sample resulted in a unique interspace profile, representing the amplified interspace fragments. Profiles consist of vectors containing three variables: fragment length, fragment abundance (measured in relative fluorescent units, RFU), and fluorescent color, representing the associated phylum group of each fragment. For whole-profile correlations, all RFU abundance values were log2 transformed.

#### Phylum and species abundance

A boxplot was made for abundance at the phylum level for Proteobacteria, Bacteroidetes, and Firmicutes/Actinobacteria/Fusobacteria/Verrucomicrobia (FAFV group). *P*-values for differences in abundance of the SDD group versus the control group were calculated for all phyla, by performing a two-sample *t*-test. A *p*-value of < 0.01 was considered statistically significant.

#### UPGMA clustering analysis

A clustered heat map analysis was made by generating a correlation matrix based on cosine correlation of all log2 transformed profile data, followed by unsupervised clustering with the unweighted pair group method with arithmetic mean (UPGMA).

#### Diversity analysis

To characterize species diversity in a sample, Shannon diversity index was calculated. Shannon’s diversity index accounts for both abundance and evenness of a species present in a sample by calculating the proportion of species relative to the total number of species [15]. Diversity was calculated for the overall composition and per phylum, based on the resulting profiles using the R 2.15.2 software package. *P*-values for difference in Shannon’s diversity index of the SDD group versus the control group were calculated, by performing a two-sample *t*-test. A *p*-value of < 0.05 was considered statistically significant.

#### Partial least squares discriminant analysis

PLS-DA is a supervised pattern recognition technique, used in cases where the number of independent variables (species in microbiota analysis) may be larger than the number of data points (samples). It aims to identify patterns in complex, high dimensional data by rotating PCoA (principal components analysis) components, such that a maximum separation among classes is obtained and to understand which variables carry the class separating information. PLS-DA was performed to provide a quantitative estimate of the discriminatory power of each OTU. The discriminatory power of each OTU is expressed as a variable importance value, which we used to define the OTU’s that were the most discriminant between the SDD group and the control group. A list of the four most discriminant species was made.

### Role of the funding source

The sponsor of this study had no role in the study design, in the collection, analysis, and interpretation of data, nor in the writing of the report.

## Results

One hundred and eighteen patients underwent a rectal swab for microbiota analysis. Fifty-six patients received SDD and 62 patients were allocated to the control group. The patients’ baseline characteristics are shown in Table 1.

**Table 1:** Baseline characteristics

	SDD (*n* = 56)	Control (*n* = 62)
Age (years)	66.7 (7.9)	69.2 (9.7)
BMI (kg/m^2^)	26.0 (3.6)	25.8 (4.2)
Sex (M:F)	34 : 22	39 : 23
ASA fitness grade		
I (healthy)	17	19
II (mild systemic disease)	35	33
III (severe systemic disease)	4	10
Diabetes (y/n)	8/45	6/56
Missing	3	0
Kidney disease (y/n)	1/52	4/52
Missing	3	6
Auto-immune disease (y/n)	2/51	2/60
Missing	3	0
Active smoker (y/n)	6/45	3/53
Missing	5	6
Neoadjuvant therapy (y/n)	5/51	8/54
Surgical intervention		
Right hemicolectomy	17	20
Rransverse colon resection	1	1
Left hemicolectomy	5	5
Sigmoidresection	15	17
Low anterior resection	17	18
Subtotal colectomy	1	1
Tumor type (adenocarcinoma/other)	56/0	62/0
Tumor stage		
I	17	17
II	15	22
III	24	23

Values in parentheses are standard deviations. *BMI*, body mass index; *ASA*, American Society of Anesthesiology

**Fig. 1:**
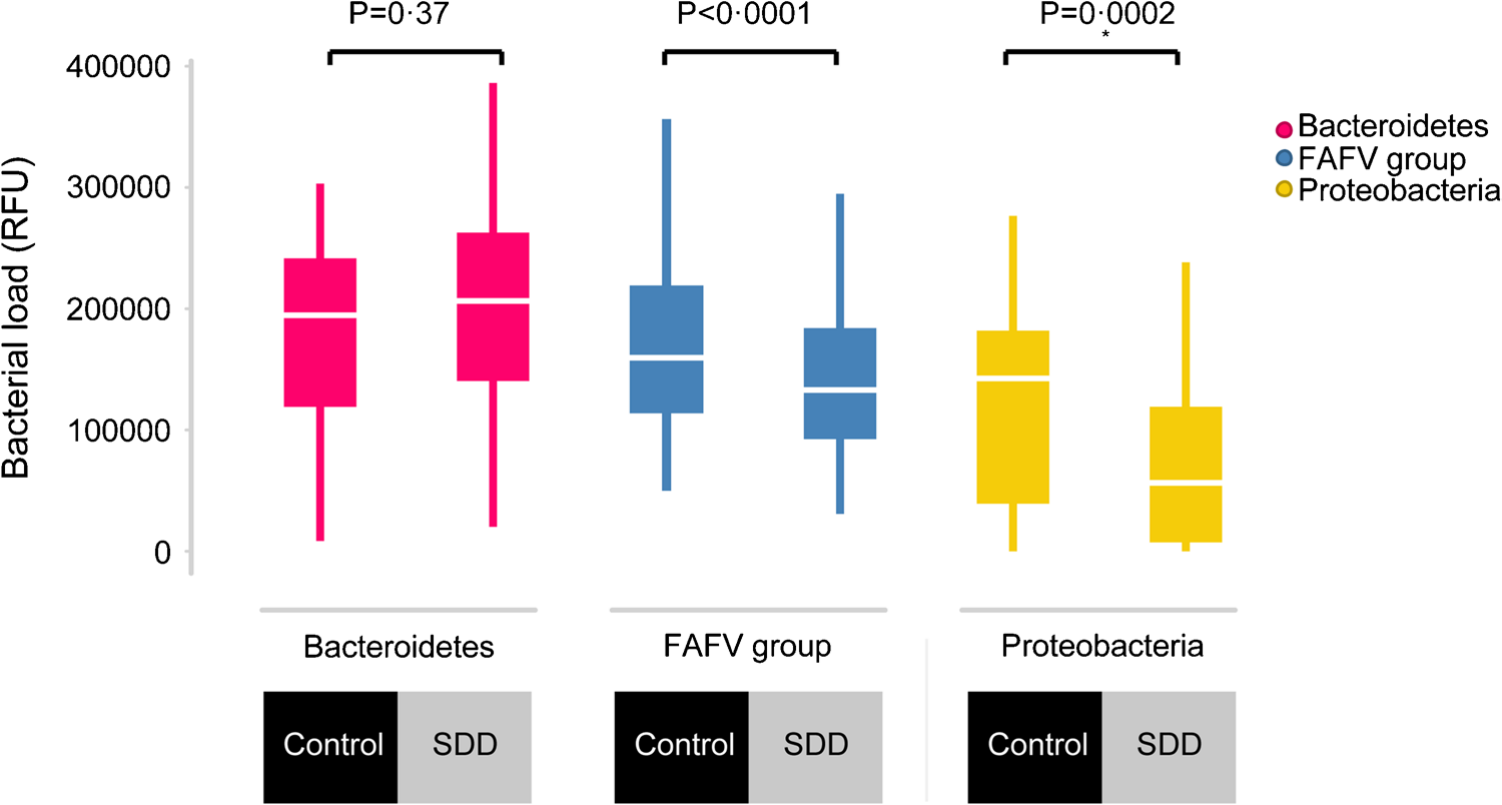
Phylum abundance analysis. Boxplot of the phylum abundance analysis. Total Proteobacteria and FAFV group abundance was significantly reduced in the SDD group versus the control group (*P* = 0.0002, respectively *P*<0.0001)

Total abundance of the phylum Proteobacteria (*P* = 0.0002) and the FAFV group (*P* < 0.0001) was significantly decreased in the SDD group, compared to the control group (Fig. 1). Total abundance of the phylum Bacteroidetes was not different between the SDD group and the control group (*P* = 0.37).

Shannon diversity was significantly decreased in the SDD group, compared to the control group. This was observed for all phyla combined (*P*= 0·0081), but was mainly defined by a decreased diversity of the phyla Proteobacteria (*P*= 0·047) and FAFV group (*P*< 0.0001).

**Fig. 2:**
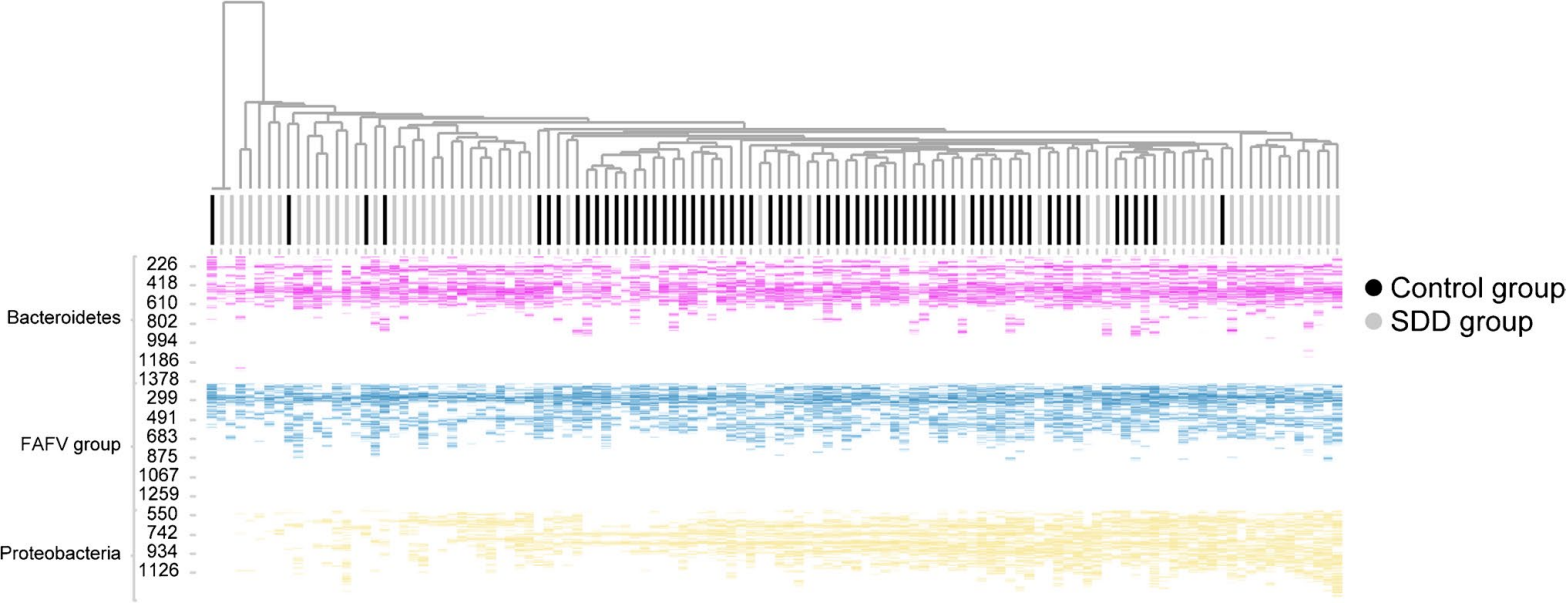
UPGMA clustering analysis. Unsupervised clustering based on Proteobacteria, depicted as a heat map of all microbiota profiles per patient, colored by phylum. A clear subclustering into two subgroups for SDD is shown. One SDD subgroup shows reduction of Proteobacteria, compared to controls. And one SDD subgroup showed no clear reduction in Proteobacteria, but a shift in Proteobacteria composition

A heat map was generated from all IS-profiles, based on clustering by Proteobacteria. There was no clear clustering based on Bacteroidetes or the FAFV group. Based on Proteobacteria, there was a clear separation of the SDD group from the control group (Fig. 2).

**Fig. 3:**
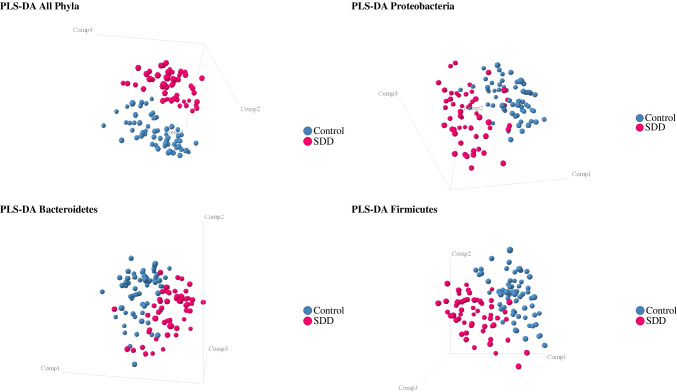
Partial least squares discriminant analysis for all phyla, Proteobacteria, Bacteroidetes, and the FAFV group. PLS-DA showed clear separation of SDD samples versus control samples, based on total microbiota analysis

**Fig. 4:**
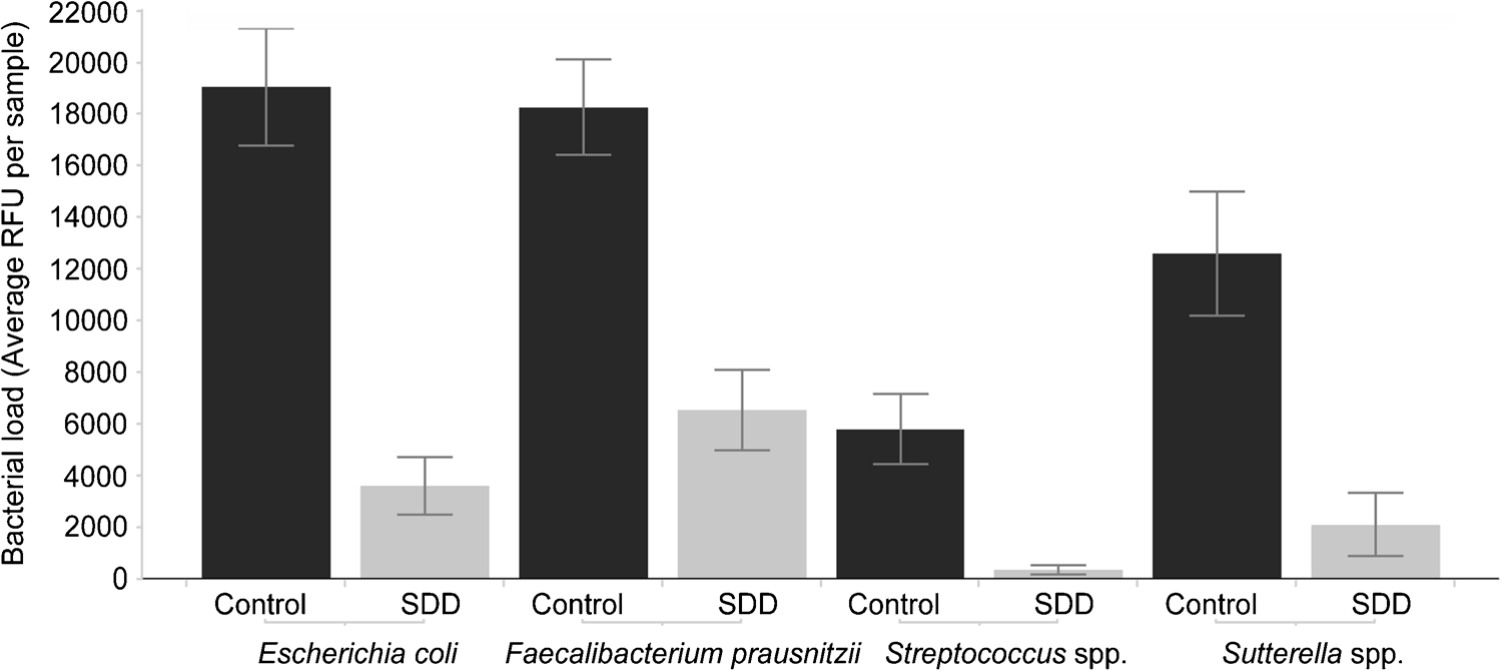
Abundance bar chart of the 4 species, discriminating most between SDD and control samples. Samples of the SDD group were associated with a decreased abundance of *E. coli*, *F. prausnitzii*, *Sutterella* spp., and *Streptococcus* spp.

Supervised classification with PLSDA showed a clear separation of gut microbial profiles between the SDD group and the control group (Fig. 3). The clearest separation was found when all phyla were taken together (sensitivity, specificity: 92%, 91%), with an accuracy of 92%. Samples of the SDD group were associated with a decreased abundance of *E. coli*, *Sutterella* spp., *Faecalibacterium prausnitzii*, and *Streptococcus* spp (Fig. 4), as defined by PLS-DA.

Although, based on Proteobacteria, there was no clear separation of the SDD group from the control group, the SDD group clustered into two separate groups. One group with a reduction in Proteobacteria abundance and diversity (hereafter referred to as SDD no Proteobacteria group) and one group without a clear reduction in Proteobacteria, but a shift in Proteobacteria composition (hereafter referred to as SDD shift Proteobacteria group) (Fig. 2). A Random Forest supervised classification analysis was performed on 21 samples of the SDD shift Proteobacteria group and 22 samples of the control group, in order to assess the most discriminant Proteobacteria between these groups to determine which species contributed most to the shift. We found that in the SDD shift Proteobacteria group, the shift was mostly defined by a decrease in *Escherichia coli* and *Sutterella* spp., and an increase in *Desulfovibrio* spp. and *Hafnia alvei*, compared to the control group.

## Discussion

This study shows that SDD not only decreases colonization of the gastrointestinal tract with potential pathogenic Gram-negative microorganisms, but also reduces the abundance of normal colonizers of our gastrointestinal system and leads to a shift in total microbiota composition. Furthermore, administering SDD does not invariably lead to the desired decrease in potential pathogenic Gram-negative bacteria in every patient.

On the phylum level, we found that SDD led to a significant decrease in abundance and diversity of the phylum Proteobacteria, to which the targeted potentially pathogenic Gram-negative bacteria belong [16]. In line with this finding, a decrease in abundance of *E. coli* was found, one of the most common representatives of the Gram-negative potential pathogens and also a representative of the phylum Proteobacteria. This is in line with previous studies, showing a decrease in abundance of Gram-negative bacteria including *E. coli* [1, 7]. It is also in line with expected activity spectrum of colistin and tobramycin, two of the three components of SDD.

The abundance and Shannon diversity of the FAFV group was also significantly decreased in the SDD group, compared to the control group. Within the FAFV group, the abundance of *F. prausnitzii*, which is regarded as an important commensal and potentially beneficial species, was significantly diminished in the SDD group, compared to the control group. Previous studies have also shown that SDD affects the abundance of *F. prausnitzii*. Benus et al. have shown that *F. prausnitzii*, *Eubacterium rectale*, and *Roseburia intestinalis* abundance is significantly decreased in SDD patients [7]. These bacteria belong to the *Clostridium* cluster IV and XIVa group, which were also shown to be reduced as a result of SDD in studies performed by Buelow et al. [8, 17]. *Faecalibacterium* is one the most abundant genera in the gut [18]. The decrease in *Faecalibacterium prausnitzii* explains the decrease in the FAFV group for a significant part. *Faecalibacterium prausnitzii* is difficult to culture and is extremely sensitive to oxygen [19].

Very little is known about susceptibility to antibiotics of this species, and no clinical breakpoints for antibiotics have been set. Nevertheless, Benus et al. have shown in vitro susceptibility of *F. prauznitzii* to tobramycin, by performing susceptibility testing for colisitin and tobramycin on *F. prausnitzii*. They have found that the MIC of tobramycin for *F. prausnitzii* was 4 µg/ml. In SDD, per dose, 60 mg of tobramycin is administered, suspended in 10 ml of water. With each administration, 60 mg of tobramycin is distributed in 2 l of gastrointestinal fluid, so the concentration of tobramycin is estimated to be approximately 30 µg/ml. The MIC of 4 µg/ml should easily be attained. Our study might indicate susceptibility of *F. prauznitzii* to tobramycin in vivo.

Additionally, we have found that SDD significantly decreases *Streptococcus* spp. abundance, despite the fact that colistin and tobramycin have no in vitro activity against *Streptococcus* spp. Some *Streptococcus* spp. have immunomodulatory properties and play an important role in shaping the immune system and immune homeostasis [20]. Previous studies have characterized the interspecies interaction between Gram-negative bacteria and streptococci. Gram-negative bacteria have been shown to promote streptococcal colonization in the airway of Cystic fibrosis patients, and to promote streptococcal biofilm formation [21, 22]. Hence, in vitro antibiotic susceptibility of a bacterium may have a different or contrasting effect in vivo, because the micro-organism is part of a complex polymicrobial environment and interacts with other species [23].

These complex microbial interactions may partly explain why the whole microbial community is affected by SDD, as shown in our study. Not only are specific taxonomic groups affected by SDD, but the microbiota composition as a whole is affected by SDD. Based on supervised microbiota analysis with PLS-DA, there was a clear separation between the SDD group and the control group, based on all phyla taken together. Buelow et al. also found that microbiota profiles from healthy patients were clearly distinct from microbiota profiles from patients receiving SDD during ICU stay [17].

To our knowledge, no supervised classification technique has yet been used to obtain the discriminatory power of each OTU in defining the microbial difference between patients treated with SDD and controls. The most discriminatory species between the SDD group and the control group were *E. coli*, *Sutterella* spp., *F. prausnitzii*, and *Streptococcus* spp. With unsupervised UPGMA clustering, there was a separation of control samples from SDD samples. The SDD samples were further divided into two separate subgroups. One subgroup showed the expected decrease in Proteobacteria, but the second subgroup showed a shift in composition of the Proteobacteria population. By means of the Random Forest technique, we determined the species within the phylum Proteobacteria that discriminated most between the control group and the SDD subgroup with a shift in Proteobacteria composition. The SDD subgroup with a shift in Proteobacteria showed a decrease in *E. coli* and *Sutterella* spp. but an increase in *Desulfovibrio* spp. and *H. alvei*. This contrasting effect of SDD has never been shown in previous studies. *H. alvei* is a Gram-negative rod with a naturally occurring resistance to colistin [24]. *Desulfovibrio* spp. are strictly anaerobic Gram-negative rods that are resistant to colistin [25]. As tobramycin has been described to be inactive in an anaerobic environment, colistin resistance alone may confer resistance to SDD [26]. The increase of *Desulfovibrio* spp. and *H. alvei* in the SDD Proteobacteria subgroup might be a reflection of selection of SDD resistant bacteria.

*Desulfovibrio* spp. have been associated with a variety of chronic inflammatory diseases, such as periodontitis and inflammatory bowel disease [27], and they have been shown to induce apoptosis of gastrointestinal epithelial cells [28]. Both *H. alvei* and *Desulfovibrio* are bacteria that can cause infections [29–32], but more importantly, their dominance over more common gut Proteobacteria during SDD administration illustrates the effect SDD can have in selecting antibiotic resistant bacteria in a complex microbial community. Abis et al. have shown that administering perioperative SDD in colorectal cancer patients, undergoing surgery, decreased the number of postoperative infectious complications [16], but our findings show that not all patients may benefit from the advantages conferred by of SDD. Interestingly, our results provide a first indication that it might be feasible to predict in which patients SDD will exert its desired effect and in which patients its use should be avoided. By performing detailed analysis of the gut microbiota prior to the administration of SDD, this possibility may be further explored.

Our study has limitations. Composition of intestinal microbiota was assessed after administering SDD. No samples were taken before adminestering SDD in the SDD group. Only differences in intestinal microbiota composition between the SDD and control group could be described. Furthermore, patients in this study where colorectal cancer patients. Colorectal cancer is associated with a change in gut microbiota composition, which could have influenced the gut microbiota composition in our patient group. However, our results extend previous results and offer novel insights into the sheer effects of the (oral component of) SDD on gut microbiota composition. Our study is unique because it is randomized and controlled, in a large sized homogenous patient group. This offers the opportunity to get a better insight in the isolated effect of SDD than is possible in a highly heterogeneous intensive care unit population. Moreover, as a first, we have used rectal swabs to analyze the effect of SDD on gut microbiota composition. It has been previously shown that microbiota composition, derived from rectal swab samples, is similar to that from fecal samples [13]. However, rectal swabs have the additional advantage that they can be taken on demand, enabling routine monitoring of microbiota in a clinical setting.

Although administering SDD has been proven to reduce ICU related mortality, and postoperative infectious complications in patients with colorectal cancer surgery, we postulate that SDD leads to a difference in intestinal microbiota signature in comparison to the intestinal microbiota in patients in a comparable control group who received no SDD. A signature that not only shows differences in the microbiota targeted by SDD, but also in the beneficial bacteria. Furthermore, the desired effect of SDD is not always attained, but a shift towards antibiotic resistant Proteobacteria may be found. Early identification of patients at risk for showing this reaction may assist in reducing infection risk and increasing the effectivity of SDD. Additional longitudinal studies are needed to address this question.

## Data Availability

Not applicable.
